# Perceived stress by mototaxi drivers and its relationship with sociodemographic and occupational characteristics

**DOI:** 10.1590/0034-7167-2022-0505

**Published:** 2023-10-09

**Authors:** Lídia Cíntia Silva Cidreira, Jules Ramon Brito Teixeira, Fernanda Carneiro Mussi

**Affiliations:** I Universidade Federal da Bahia. Salvador, Bahia, Brazil; II Universidade Estadual de Feira de Santana. Feira de Santana, Bahia, Brazil

**Keywords:** Motorcycles, Stress, Occupational Health, Working Conditions, Men’s Health, Motocicletas, Estrés, Salud del Empleado, Condiciones de Trabajo, Salud del Hombre, Motocicletas, Estresse, Saúde do Trabalhador, Condições de Trabalho, Saúde do Homem

## Abstract

**Objective:**

To investigate the association of sociodemographic and occupational characteristics with a high level of perceived stress in motorcycle taxi drivers.

**Method:**

Cross-sectional study carried out with motorcycle taxi drivers who answered instruments on sociodemographic and occupational variables - Perceived Stress Scale, Job Content Questionnaire and Effort-Reward Imbalance. Descriptive statistics, Pearson’s chi-square test and Poisson regression with robust variance were used. Statistical significance was 5%.

**Results:**

Of the 800 motorcycle taxi drivers, 46.8% had a high level of perceived stress. In the multivariate analysis, a high level of stress was associated with low control over work (PR=7.76; 95%CI=5.19-11.61), low social support at work (PR=3.87; 95%CI =2.95 5.08), working hours longer than eight hours a day (RP=1.47; 95%CI=1.21-1.78) and monthly income less than or equal to two minimum wages (PR=1.34;95%CI=1.13-2.58).

**Conclusion:**

Long working hours, occupational stressors and low income were associated with a high level of perceived stress. Public policies and interventions to minimize occupational stressors are essential.

## RESUMO

**Objetivo:**

Investigar associação de características sociodemográficas e ocupacionais com alto nível de estresse percebido em mototaxistas.

**Método:**

Estudo transversal realizado com mototaxistas que responderam a instrumentos sobre variáveis sociodemográficas e ocupacionais - *Perceived Stress Scale, Job Content Questionnaire* e *Effort-Reward Imbalance*. Empregou-se a estatística descritiva, teste qui-quadrado de Pearson e regressão de Poisson com variância robusta. A significância estatística foi 5%.

**Resultados:**

Dos 800 mototaxistas, 46,8% apresentaram alto nível de estresse percebido. Na análise multivariada, o alto nível de estresse foi associado com baixo controle sobre o trabalho (RP=7,76; IC95%=5,19-11,61), baixo suporte social no trabalho (RP=3,87; IC95%=2,95 5,08), jornada de trabalho maior que oito horas por dia (RP=1,47; IC95%=1,21-1,78) e renda mensal menor ou igual a dois salários mínimos (RP=1,34; IC95%=1,13-2,58).

**Conclusão:**

Extensa jornada de trabalho, estressores ocupacionais e baixa renda foram associados ao alto nível de estresse percebido. Políticas públicas e intervenções para minimizar estressores ocupacionais são imprescindíveis.

## RESUMEN

**Objetivo:**

Investigar relación de características sociodemográficas y ocupacionales con alto nivel de estrés percibido en mototaxistas.

**Método:**

Estudio transversal realizado con mototaxistas que respondieron a instrumentos sobre variables sociodemográficas y ocupacionales - *Perceived Stress Scale, Job Content Questionnaire* y *Effort-Reward Imbalance*. Empleado la estadística descriptiva, prueba chi-cuadrado de Pearson y regresión de Poisson con varianza robusta. La relevancia estadística fue 5%.

**Resultados:**

De los 800 mototaxistas, 46,8% presentaron alto nivel de estrés percibido. En el análisis multivariado, el alto nivel de estrés fue relacionado con bajo control sobre el trabajo (RP=7,76; IC95%=5,19-11,61), bajo soporte social en el trabajo (RP=3,87; IC95%=2,95 5,08), jornada laboral mayor que ocho horas al día (RP=1,47; IC95%=1,21-1,78) y renta mensual menor o igual a dos salarios mínimos (RP=1,34; IC95%=1,13-2,58).

**Conclusión:**

Extensa jornada laboral, estresores ocupacionales y baja renta fueron relacionados al alto nivel de estrés percibido. Políticas públicas e intervenciones para minimizar estresores ocupacionales son imprescindibles.

## INTRODUCTION

Mototaxi driving is a regulated occupation since 2009 in Brazil, existing in most cities, especially in small and medium-sized ones^(1)^. This work is carried out predominantly by men and has become advantageous and attractive as it allows for the management of one’s own business, giving the option for workers to be easily located at various points throughout the city and offering a source of income for personal and family support^(2)^.

Despite the job opportunities generated by mototaxis, these workers are subject to precarious working conditions^(3-5)^, which can predispose to the emergence and maintenance of high levels of stress. These working conditions include urban violence and environmental adversities, such as traffic, noise, climatic variations, pollution, among others^(6)^. Working in traffic is, in itself, a stressor and can cause fatigue, anxiety, depression, phobias, cardiovascular diseases, aggression, gastrointestinal and musculoskeletal problems, back and neck pain, among other problems^(7)^.

Among the precarious working conditions that may be related to stress, the characteristics of the workday of motorcycle taxi drivers stand out, such as the intense pace, six to seven days a week and eight hours or more a day, often with amendments of shifts and without a break or an appropriate place to rest^(6-7)^. The intense and repetitive daily work routine in a precarious environment, in addition to being a source of stress, can affect health in general, productivity and job satisfaction^(6)^. The persistence of work in this routine can increase the level of perceived stress and cause physical and/or psychological changes, which determine the progressive decline of health conditions and quality of life^(2-5,7)^ and the temporary or permanent removal from work. work activities^(3-4)^.

Motorcycle taxi drivers are also exposed to the psychosocial aspects of work, known as occupational stressors, which include control over the tasks performed, psychological demand, social support at work^(8-9)^ and excessive commitment^(10-13)^, among others. These stressors can be considered potential risk factors for the appearance of psychological and/or physical pathologies in workers^(6)^. From this perspective, there is evidence that the motorcycle taxi drivers’ work is carried out in a situation of high demand, which combines high psychological demand and low control over the activities carried out and has a negative impact on the perception of quality of life of these workers^(4-6)^.

The characteristics of motorcycle taxi drivers’ working hours and occupational stressors may also favor the adoption of unhealthy lifestyles, such as the lack of specific times for meals, sleep and rest; having meals on the street, encouraging the consumption of high-calorie and ultra-processed foods; lack of time or inclination for leisure-time physical activity; permanence for excessive time in sedentary behavior, exposure to smoking^(14)^; and excessive alcohol consumption^(15)^. Thus, occupational activity and its interaction with environmental, behavioral and genetic factors may imply a higher level of stress and constitute predictors of cardiovascular risk^(3,14,16)^.

There is still a lack of knowledge in the literature about the perceived level of stress in motorcycle taxi drivers and its relationship with sociodemographic and occupational characteristics, including, in the latter, the variables of working hours and occupational stressors. The few studies involving this class of workers explored the occurrence of traffic accidents^(14,17-19)^ or focused on occupational stressors as the main exposure to outcomes such as quality of life, ability to work and level of physical activity^(3-5)^.

It is worth noting that this group of workers is mostly made up of men. They, due to the social constructions of being a man, find it difficult to verbalize what they feel, demonstrate weakness, and dedicate themselves to taking care of themselves. In Brazilian society, there is still a culture that they should be strong, invulnerable and play the role of family provider^(20)^. The culture of masculinity highlights the need for actions to promote health, prevention, and control of cardiovascular risk factors in motorcycle taxi drivers, with a focus on gender issues, care planning and reduction of disabilities and mortality from cardiovascular diseases.

The identification of factors associated with high levels of perceived stress in motorcycle taxi drivers can guide the implementation of public policies and practices in health and nursing care aimed at promoting health and protecting this working class. It should be noted that the positive result of planning actions to provide health and safety services in the workplace developed by occupational nursing is essential to offer greater safety at work, better performance and minimization of absenteeism and improvement in the quality of life of the worker. The study encourages discussion of the occupational hazards to which motorcycle taxi drivers are exposed and that can affect the quality of life and work ability of these workers, as well as the right to ensure decent working conditions.

## OBJECTIVE

To investigate the association of sociodemographic and occupational characteristics with the high level of perceived stress in motorcycle taxi drivers.

## METHODS

### Ethical aspects

The study was approved by the Research Ethics Committee and met the recommendations for national and international research with human beings.

### Study design, period, and place

Cross-sectional study, part of the matrix project entitled “Factors associated with overweight and cardiovascular risk in motorcycle taxi drivers”, which was carried out with motorcycle taxi drivers in the municipality of Jequié, state of Bahia (BA), Brazil, from January to October 2017. The present study was constructed according to the Strengthening the reporting of observational studies in epidemiology (STROBE) tool.

### Sample

The sample consisted of 800 motorcycle taxi drivers chosen for convenience due to the informal nature of the work they perform. Inclusion criteria were working as a motorcycle taxi driver for at least one year, being at least 21 years old and being male. Motorcycle taxi drivers with another job or occupation, using medication to lose weight and undergoing bariatric surgery in the year prior to data collection were excluded. In the sample calculation, the expected prevalence of 50% was adopted for the outcome, precision of 5%, confidence level of 95% and power of 80%, additional 20% for losses, reaching a sample of 398 motorcycle taxi drivers. The calculation aimed to identify whether the sample obtained for the matrix project had the power to detect the intended associations in this study.

### Study protocol

Data was collected by two doctoral students in Nursing and a nurse, duly trained. The motorcycle taxi drivers were approached at the motorcycle taxi ranks, located on the streets of the city of Jequié - BA, when they were waiting for the service request. Compliance with the inclusion and exclusion criteria was verified and participation in the study was requested. The reading, clarification and signing of the Free and Informed Consent Form were carried out. Data were obtained using four instruments from the matrix project, included in a free access digital platform (Google Forms) and installed on a tablet for application during the interview.

The dependent variable was the level of perceived stress, measured by the Perceived Stress Scale (PSS-10), developed by Cohen^(21)^. Originally with 14 items, the short version of ten items was cross-culturally adapted^(22)^ and validated in Brazil for different age groups^(23)^. The items consist of a five-point Likert response scale: 0 = never; 1 = almost never; 2 = sometimes; 3 = almost always; to 4 = always. The questions have a positive or negative connotation, and the positive ones should have the score inverted. The score is estimated by the sum of the scores, ranging from 0 to 40^(21-22)^. Initially perceived stress levels were categorized as: low (0 to 13 points); moderate (14 to 26 points); and high (27 points or more). For the analyses, this variable was dichotomized into: 0 - Low/Moderate; and 1 - High level of stress^(24)^.

The independent variables were sociodemographic characteristics (age, marital status, education, self-reported race/skin color and motorcycle taxi driver’s monthly income in minimum wages) and occupational characteristics related to working time as a motorcycle taxi driver (in years), working hours (number of working days per week, number of working hours per day, number of work shifts per day and rest breaks) and occupational stressors (control over work, psychological demand, social support at work and overcommitment to work). These variables were raised by a sociodemographic and occupational survey made up of closed and semi-structured questions. Occupational stressors were investigated using the instruments described below.

Job Content Questionnaire (JCQ): the validated version for formal and informal workers in Brazil was applied^(9)^. Among the dimensions of the JCQ, the following were evaluated: psychological demand (8 items), control over work (9 items) and social support at work (5 items). The questions are made up of a four-point Likert scale, with scores ranging from 1 (strongly disagree) to 4 (strongly agree). The score for each dimension was estimated by adding the scores received on the questions and dichotomized into low level (less than or equal to the median) and high level (greater than the median).

Effort-Reward Imbalance: designed by Siegrist^(10)^ and adapted to Brazilian Portuguese for workers^(11)^, the instrument assesses three dimensions: effort (6 items), reward (11 items) and overcommitment to work (6 items). In this study, only excessive commitment to work was analyzed, which reflects an investment of greater effort by the worker with the objective of approval and better esteem^(11-12)^. The items are composed of a four-point Likert scale (strongly agree to strongly disagree). Excessive commitment was dichotomized into “absent” and “present”, using the median cut-off point^(25)^.

### Analysis of results and statistics

The database was organized using the Statistical Package for the Social Sciences (SPSS) software, version 20.0; and the analysis was processed in Stata, version 15. In the bivariate analysis, Pearson’s chi-square test was used, with estimation of prevalence and crude prevalence ratio (PR), with their respective 95% confidence intervals. The variables that presented p ≤ 0.20 in the bivariate analysis were included in the multiple analysis, performed using the Poisson regression model with robust variance. The backward procedure was adopted and, to choose the best model, the Akaike Information Criterion. Statistical significance was 5%. Multicollinearity was ruled out by analyzing the mean VIF and individual variables less than 10.

## RESULTS

The study sample consisted of 800 motorcycle taxi drivers, of which almost half had a high level of perceived stress (46.8%) (Table 1).

**Table 1:** Distribution of motorcycle taxi drivers (N = 800) according to perceived stress level, sociodemographic and occupational characteristics, Jequié, Bahia, Brazil, 2017

Variable	n	%
Perceived stress level		
Low	188	23,5
Moderate	238	29,7
High	374	46,8
Sociodemographic characteristics		
Age group		
Up to 35 years	462	57,8
≥ 36 years	338	42,2
Educational level		
Elementary School	272	34,0
High school/Higher education	528	66,0
Marital status		
No partner	297	37,1
With partner	503	62,9
Self-reported race/skin color		
Black	472	59,0
Non-black	328	41,0
Mototaxi driver monthly income^*^		
> 2 minimum wages	672	84,0
≤ 2 minimum wages	128	16,0
Occupational characteristics		
Workday		
Working time in years		
≤ 7 years	461	57,6
> 7 years	339	42,4
Working days/week		
≤ 5 days	267	33,38
6-7 days	533	66,63
Working hours/day		
≤ 8 hours	218	27,3
> 8 hours	582	72,7
Work shifts/day		
Diurnal	482	60,3
Nocturnal	318	39,7
Rest break		
Yes	365	45,6
No	435	54,4
Occupational stressors		
Control over work		
High	327	40,9
Low	473	59,1
Psychological demand		
High	505	63,1
Low	295	36,9
Social support at work		
High	342	42,8
Low	458	57,2
Overcommitment to work		
Absent	391	48,9
Present	409	51,1

* Minimum wage value at the time of data collection: R$ 937.00.

As for sociodemographic variables, 57.8% were aged up to 35 years, 66.0% had high school or higher education, 62.9% lived with a partner, 59.0% self-declared as black race/color and 84,0% received more than two minimum wages per month (Table 1).

Regarding occupational characteristics, regarding working hours, the majority had been motorcycle taxi drivers for seven years or less (57.6%), worked six to seven days a week (66.6%), workload eight hours a day or more (72.7%), worked the day shift (60.3%) and without a break for rest (54.4%).

Related to occupational stressors, it was observed that 59.1% of motorcycle taxi drivers had low control over their work (median = 57.9); 63.1% had high psychological demand (median = 34.9); 57.2% low social support at work (median = 7.4); and 51.1%, excessive commitment to work (median = 17.0) (Table 1).

In the bivariate analysis, regarding sociodemographic variables, motorcycle taxi drivers aged up to 35 years had a prevalence of high stress levels 34.0% lower than those aged 36 years or more (PR = 0.66; CI95% = 0.57-0.76); and those with primary education had a 69.0% higher prevalence compared to those with secondary/higher education (PR = 1.69; 95%CI = 1.46-1.94). In addition, motorcycle taxi drivers without a partner had a 24.0% higher prevalence of high levels of stress compared to those who did not (PR = 1.24; 95%CI = 1.06-1.47), and those of race/ blacks had a 43.0% higher prevalence than non-blacks (PR = 1.43; 95%CI = 1.21-1.69) (Table 2).

**Table 2:** Prevalence of high stress level in motorcycle taxi drivers (N = 800) according to sociodemographic and occupational characteristics, Jequié, Bahia, Brazil, 2017

Variable	n	P (%)	*p* ^^*^^	PR	CI95%
Sociodemographic characteristics					
Age group					
≥ 36 years (n = 338)	196	58.0	**< 0.001**	1.00	-
Up to 35 years (n = 462)	178	38.5		0.66	0.57-0.76
Educational level					
High school/Higher education (n = 528)	200	37.8	**< 0.001**	1.00	-
Elementary School (n = 272)	174	63.9		1.69	1.46-1.94
Marital status					
With partner (n = 503)	254	50.5	**< 0.007**	1.00	-
Without partner (a) (n = 297)	120	40.4		1.24	1.06-1.47
Self-reported race/skin color					
Non-black (n = 328)	122	37.2	**< 0.001**	1.00	-
Black (n = 472)	252	53.4		1.43	1.21-1.69
Mototaxi driver monthly income					
> 2 minimum wages (n = 672)	322	47.9	**< 0.001**	1.00	-
≤ 2 minimum wages (n = 128)	52	40.6		0.84	0.67-1.06
Occupational characteristics					
Workday					
Working time in years					
≤ 7 years (n = 461)	153	33.2	**< 0.001**	1.00	-
> 7 years (n = 339)	221	65.1		1.96	1.68-2.28
Number of working hours per day					
≤ 8 hours (n = 218)	44	20.1	**< 0.001**	1.00	-
> 8 hours (n = 582)	330	56.7		2.80	2.13-3.69
Rest break					
Yes (n = 365)	88	24.1	**< 0.001**	1.00	-
No (n = 435)	286	65.7		2.72	2.24-3.31
Occupational stressors					
Control over work					
High (n = 327)	22	6.7	**< 0.001**	1.00	-
Low (n = 473)	352	74.4		11.06	7.36-16.62
Psychological demand					
High (n = 295)	130	44.7	0.251	1.00	-
Low (n = 505)	244	48.3		1.09	0.93-1.28
Social support at work					
High social support (n = 342)	41	11.9	**< 0.001**	1.00	-
Low social support (n = 458)	333	72.7		6.06	4.52-8.12
Overcommitment to work					
Absent (n = 391)	157	40.1	**< 0.001**	1.00	-
Present (n = 409)	217	53.0		1.32	1.13-1.53

* p obtained by Pearson's chi-square test; P - Prevalence; PR - prevalence ratio; 95% CI - 95% confidence interval.

As for occupational characteristics, there was a 96.0% higher prevalence of high stress level in motorcycle taxi drivers with more than seven years of work compared to those with seven or less years (PR = 1.96; CI95% = 1.68-2.28). Motorcycle taxi drivers working more than eight hours a day had a prevalence 2.8 times higher for high levels of stress compared to those who worked eight hours or less (PR = 2.80; 95%CI = 2.13-3.69) , and those who did not take a break during the workday had a prevalence almost three times higher compared to those who did (PR = 2.72; 95%CI = 2.24-3.31) (Table 2).

With regard to occupational stressors, the prevalence of high levels of stress was 11 times higher in motorcycle taxi drivers with low control at work than with high control (PR = 11.06; 95%CI = 7.36-16.62), six times higher in motorcycle taxi drivers with low social support than with high social support at work (PR = 6.06; 95%CI = 4.52-8.12) and 32% higher for those who had excessive commitment to work compared to those who did not have it (PR = 1.32; 95%CI = 1.13-1.53).

In the bivariate analyses, only the psychological demand did not present a statistically significant association with the high level of stress (p = 0.251), (Table 2).

In the multivariate analysis, variables such as rest breaks (p = 0.593), overcommitment to work (p = 0.069) and working time (p = 0.053) were excluded from the modeling because they did not meet the statistical significance criterion and because of the exclusion did not result in a decrease in the value of the AIC.

In the adjusted model, the factors that most contributed to the high level of stress were: low control over work (PR = 7.76; 95%CI = 5.19-11.61), low social support at work (PR = 3 .87; 95%CI = 2.95-5.08), working hours longer than eight hours a day (PR = 1.47; 95%CI = 1.21-1.78) and monthly income less than or equal to two minimum wages (PR = 1.34; 95%CI = 1.13-2.58) (Table 3). The highest VIF value was observed for the variable working hours per day (3.98); and the lowest, for monthly income (1.25) (data not shown in tables). The analysis of individual and mean VIF (2.45) (Table 3) ruled out the presence of multicollinearity.

**Table 3:** Prevalence ratios and confidence intervals (95%) of the association of sociodemographic and occupational characteristics with the high level of perceived stress in motorcycle taxi drivers (N = 800), Jequié, Bahia, Brazil, 2017

Variable	PR	CI95%
Sociodemographic characteristics		
Age group		
36 years or more	1.00	-
Up to 35 years	1.13	1.05-1.22
Educational level		
High school/Higher education	1.00	-
Elementary School	1.09	1.00-1.18
Self-reported race/skin color		
Nonblack	1.00	-
Black	1.10	1.01-1.19
Taxi driver monthly income		
> 2 minimum wages	1.00	-
≤ 2 minimum wages	1.34	1.13-1.58
Occupational characteristics		
Workday		
Number of working hours per day		
≤ 8 hours	1.00	-
> 8 hours	1.47	1.21-1.78
Occupational stressors		
Control over work		
High	1.00	-
Low	7.76	5.19-11.61
Social support at work		
High	1.00	-
Low	3.87	2.95-5.08
AIC	1.23	
VIF mean	2.45	

PR - prevalence ratio; 95% CI - 95% confidence interval.

## DISCUSSION

There was a prevalence of 46.8% for a high level of perceived stress among motorcycle taxi drivers, a high estimate compared to non-professional drivers (22.3%) and lower than that observed among taxi drivers (63.3%), both from Morocco^(25)^. Brazilian and international studies on perceived stress in motorcycle taxi drivers were not identified, which made comparisons with the findings of the present study difficult.

The high level of stress perceived among motorcycle taxi drivers is a relevant finding and should be prioritized within the scope of public policies for workers’ health care, as stress is a cardiovascular risk factor^(26)^ associated with the occurrence of myocardial infarction, stroke , obesity, arterial hypertension, diabetes mellitus and smoking^(27)^. This finding endorses the need for changes in the organization of work and in the psychosocial environment of motorcycle taxi drivers, with a view to promoting health. Modifications in the physical work environment are also relevant, but it is an almost impracticable recommendation because the work is performed in traffic; and, when motorcycle taxi drivers are not transporting passengers, they stay at motorcycle taxi stands, places with precarious structure, outdoors, without protection from climate variations and adequate space for rest^(5)^.

Traffic is a potentially stressful workplace and exposes motorcycle taxi drivers to physical stressors (weather; poor road conditions; traffic jams) and social stressors (peak hours; streets crowded with pedestrians, street vendors and parked vehicles; reckless drivers). Given the continuous exposure to these stressors, emotional exhaustion and easy irritability in traffic are frequent, which contributes to the elevation of stress levels. With the difficulty of eradicating these stressors, coping approaches focused on the emotion and the problem are recommended to develop and/or strengthen positive reinforcements to control the high load of stress^(28)^.

In this study, the high level of perceived stress was associated with younger motorcycle taxi drivers, in line with the literature^(4-6)^. Younger people tend to deal with life concerns and anxiety with optimistic attitudes and a relatively positive perception of the future. However, stress is common among them, and sustaining high levels can result in difficulty in evoking compensatory mechanisms and generate deleterious effects on health, especially in the mental and cardiovascular spheres. In addition, younger workers tend to overestimate their ability to work and their abilities to deal with the strenuous workday, in order to obtain a higher income, a frequent reality among motorcycle taxi drivers^(4)^. Thus, the high level of stress among younger people may be associated with prolonged exposure to exhausting working hours, stressful life events outside of work and the inability to establish self-control mechanisms to deal with stressors.

The findings of this study related the high level of perceived stress to lower schooling in motorcycle taxi drivers. People with more education can better understand the risks and dangers and practice healthy strategies to deal with stressors^(29)^ and, in general, are better equipped with resources and well-being to deal with stress^(30)^. More educated individuals may be more likely to engage in cognitively stimulating activities, have better economic conditions, eat healthier and spend more time in leisure-time physical activities^(29)^, which are resources that contribute to stress reduction. Thus, it is important for motorcycle taxi drivers with less education, perceiving higher levels of stress, to be encouraged to develop coping skills, especially in the absence of the aforementioned protection factors. For this, health education strategies are essential and must be developed by nurses.

In this research, there was an association between a high level of perceived stress and race/black skin color. The study was carried out in a region predominantly inhabited by people of African descent^(31)^, and the black population is often the target of discriminatory acts, in addition to having lower family income and educational performance, experiencing social isolation and worse opportunities for access to the formal job market^(32)^, which are factors traditionally associated with high levels of stress.

The association identified between lower monthly income and a high level of stress corroborates a study with other population groups^(33)^. People with lower income generally have a low socioeconomic level, which is combined with a situation of lower education and worse occupational situation, factors pointed out in the literature as determinants of the high level of stress^(33)^. It should also be considered that motorcycle taxi drivers with lower incomes are more vulnerable, including due to the low perception of risk arising from their lower educational level, and may not have enough income to acquire the necessary resources to resolve the deleterious effects of stress.

The finding of a high level of stress in motorcycle taxi drivers associated with a higher number of hours worked is consistent with a population-based study in South Korea^(34)^. Long working hours are associated with an increased risk of adverse health outcomes, such as type 2 diabetes mellitus^(34)^. Their predominance and the absence of rest breaks were also documented for motorcycle taxi drivers, in an investigation in the state of Goiás^(35)^. In the impossibility of reducing the hours worked, since it impacts income, rest breaks throughout the day should be encouraged, as they can contribute to the reduction of stress levels.

The high level of stress perceived in the presence of occupational stress revealed how much the motorcycle taxi drivers’ work environment generates stress and can affect the quality of life^(4-6)^. In addition, this association highlights that occupational stress is related to the inability or decrease in the worker’s abilities to deal with demands arising from the interaction between the work environment, its content, the organizational conditions and the work capacity itself^(36)^, which in turn greatly influences perceived stress. A study with motorcyclists in Bogor identified that sources of stress at work had a positive relationship with stress symptoms, showing that the greater the occupational stress, the more stress symptoms are experienced^(37)^.

Among the occupational stressors, there was an association between a high level of perceived stress and a low level of control and social support at work. Control over work is related to the use of skills (learning new things, repetitive work, creativity, task variability and skill development) and decision-making authority (decision-making at work, influence of the work group and the managerial policy)^(38)^. Social support at work protects workers from the adverse effects of stress, helping them to redefine the problem and seek a solution^(39)^. The low level of social support has a negative impact on health and enhances the negative effect of exposure to jobs that combine a low level of control and a high level of psychological demand - which configures the high-strain work experience^(40)^.

A study that investigated associations between occupational stressors and perceived stress in elderly people in Denmark, with physically demanding jobs, showed that the lower the level of control over work and social support from co-workers, the greater the level of stress^(41)^. Research with motorcyclists in Indonesia identified that social support from family, friends or other sources reduced the stress of riding the motorcycle and the worker’s concerns, as well as favoring harmonious relationships^(42)^. Another research, which evaluated the perception of quality of life of motorcycle taxi drivers in the state of Bahia, showed that high control at work attenuates the deleterious effects of work on the psychological domain of quality of life and decreases negative feelings, anxiety, depression, occupational stress and the probability of getting sick^(6)^.

The work carried out in a situation of low control and social support, especially when associated with high psychological demand, is a more harmful experience for the health of the worker, as it generates more wear, suffering and dissatisfaction^(8)^. Some factors are evoked to explain the association between low control over work and a high level of perceived stress among motorcycle taxi drivers. The low level of control can have repercussions on a lower level of concentration when driving the motorcycle and on intercurrences in traffic, generating stress. Monotonous and repetitive work, which involves driving in traffic to transport the passenger to the destination and returning to the fixed place of work to wait for new trips, can lead to dissatisfaction and demotivation with work, which are two reactions to the stress caused by the work context^(8)^. Finally, low control may result in difficulty or inability to create positive reinforcements to control the stressful routine resulting from the daily repetitiveness of the service, work overload, time pressure and productivity^(6)^.

The job of a motorcycle taxi driver is extremely competitive, as higher income results from a greater number of trips^(6)^. Thus, as they are clustered at the point, the travel demands are distributed among the motorcycle taxi drivers, who are organized in a kind of queue, not always respected by co-workers. In this case, the more trips made by the same motorcycle taxi driver, the smaller the number of trips made by the colleague. This results in competitiveness and conflict, which is reflected in less social support between them and greater perceived stress. In addition, the absence of a supervisor reflects a loss of the protective effect from the boss, resulting in greater stress in managing work and relationships and tensions with passengers and co-workers.

It is worth mentioning that the search for the worker’s well-being is the goal of the nurse’s work. It is up to these professionals to develop actions aimed at worker safety capable of minimizing the level of perceived stress, through the promotion of health campaigns, implementation of projects, monitoring of occupational or non-occupational illnesses and their rehabilitation for the work, in addition to educational and administrative actions involving protection against chemical, physical and biological agents. The role of the nurse is even more relevant and challenging when identifying that this working class is deprived of labor rights, which, if any, would favor the implementation of health promotion, protection, and recovery actions. That said, this study reveals the urgency of public policies aimed at protecting the health of these workers.

### Study limitations

The limitations arose from the effect of the healthy worker, which excludes those on leave due to illness or disability from the workforce, making inclusion in the sample unfeasible.

### Contributions to the Area

The study guides the development of public policies and interventions to minimize job insecurity and promote a better quality of life for motorcycle taxi drivers.

## CONCLUSION

Most motorcycle taxi drivers had a high level of perceived stress associated with low control over work, low social support at work, working hours longer than eight hours a day, monthly income less than or equal to two minimum wages, self-reported race/skin color black, aged up to 35 years and less schooling.
